# Determinants of patient satisfaction with Wasfaty and community pharmacy services in Aseer, Saudi Arabia: a cross-sectional study

**DOI:** 10.3389/fmed.2026.1845392

**Published:** 2026-07-22

**Authors:** Geetha Kandasamy, Khalid Orayj, Eman Shorog, Rayah Asiri, Tahani Alanazi, Asma M. Alshahrani, Amjad Hmlan, Ayesha Siddiqua, Hanan S. Algorman

**Affiliations:** 1 Department of Clinical Pharmacy, College of Pharmacy, King Khalid University, Abha, Saudi Arabia; 2 Department of Clinical Pharmacy, College of Pharmacy, Shaqra University, Dawadimi, Saudi Arabia

**Keywords:** community pharmacy services, digital prescription system, medication availability, patient satisfaction, Saudi Arabia, Wasfaty

## Abstract

**Background:**

Wasfaty is a national electronic prescription system in Saudi Arabia linking healthcare facilities with community pharmacies to improve medication access. As digital health services expand, patient satisfaction is increasingly important, with limited evidence from the Aseer region. This study assessed patient satisfaction with Wasfaty and community pharmacy services and its associated demographic and service-related factors.

**Methods:**

A cross-sectional survey was conducted (5 September–25 October 2025) among adults (≥18 years) in the Aseer region using Wasfaty or community pharmacy services. Participants were recruited via convenience sampling and completed a structured questionnaire assessing sociodemographics, Wasfaty use, and satisfaction. Data were analyzed using descriptive and inferential statistics, with *p* < 0.05 considered statistically significant.

**Results:**

A total of 446 participants were enrolled, most of whom were female (57.6%) and aged 30–49 years (63.7%). Overall satisfaction with Wasfaty and community pharmacy services was high, with more than three-quarters reporting positive experiences. Ease of use was highly rated, while concerns were noted regarding medication availability and waiting times. Ease of use showed a moderate positive correlation with Wasfaty satisfaction (*r* = 0.48, *p* < 0.001), whereas waiting time was negatively associated (*r* = −0.21, *p* < 0.01). Multiple linear regression identified ease of use (β = 0.30), pharmacist communication (β = 0.28), and medication availability (β = 0.24) as the strongest predictors of satisfaction, while longer waiting times were negatively associated (β = −0.10, *p* = 0.02). Logistic regression showed higher odds of satisfaction among females (AOR = 4.83) and among participants reporting better pharmacist communication (AOR = 1.40), ease of use (AOR = 1.35), and medication availability (AOR = 1.28) (all *p* < 0.001).

**Conclusion:**

Overall satisfaction with the Wasfaty system and community pharmacy services was generally high, although some participants reported neutral or dissatisfied responses, indicating variability across service domains. Key concerns included medication availability, waiting times, and communication, highlighting areas for improvement to enhance patient experience and service quality.

## 1 Introduction

In recent years, the Kingdom of Saudi Arabia (KSA) has undergone substantial reforms in its healthcare system under the Vision 2030 strategic plan, aiming to enhance service delivery, meet rising demand, address chronic disease prevalence, and manage healthcare costs (1, 2). Historically, most healthcare services were provided by government facilities offering free care, while private healthcare was accessible only to individuals with insurance or the ability to pay out of pocket (1, 3). To expand access and strengthen preventive care, Vision 2030 initiatives included private community pharmacies in medication provision, increasing the private sector’s role in healthcare delivery (4). In 2018, primary and secondary government healthcare institutions implemented the Wasfaty e-prescribing system. E-prescribing is defined as “the direct computer-to-computer transmission of electronic prescriptions from the prescriber’s office to community pharmacies” (5). Through Wasfaty, patients can obtain prescribed medications from the nearest participating pharmacy. Evidence suggests that e-prescribing improves healthcare delivery by reducing costs, enhancing medication safety, and improving adherence (6). In the Saudi context, studies on Wasfaty have also reported high patient satisfaction and positive experiences with the system (7). The integration of e-prescriptions addresses challenges such as illegible handwriting and reduces the risk of prescribing errors, thereby improving patient safety (8). Wasfaty also facilitates real-time communication between pharmacies and prescribers, ensuring accurate and timely dispensing (9). For patients with chronic conditions, the system enables prescription renewal through virtual consultations, reducing the need for in-person visits and saving time for both patients and physicians (9).

Community pharmacies in Saudi Arabia are tightly regulated: only Saudi pharmacists licensed by the Saudi Commission for Healthcare Specialties may own them, with a maximum of 30 pharmacies per pharmacist. Most pharmacies are operated by one or two pharmacists, open 8–12 h daily for 6 days a week, and some function 24/7 (10–12). Oversight is provided by the Ministry of Health (13). According to Andersen’s behavioral model, healthcare utilization is influenced by environmental factors, demographic characteristics, health behaviors, and outcomes such as patient satisfaction (14). Patient dissatisfaction may reduce service utilization, compromise medication adherence, worsen health outcomes, and increase healthcare costs. Evaluating patient satisfaction with Wasfaty and community pharmacy services is therefore critical (14). Community pharmacies must meet several requirements to participate in Wasfaty, including a valid premises license, Nupco registration, use of approved suppliers, licensed pharmacists, and access to the online system with proper equipment. Currently, 3,100 community pharmacies are registered with Wasfaty, serving 2,069 primary healthcare centers and 228 hospitals across 174 cities in Saudi Arabia (15). Regional studies have highlighted opportunities for improvement. A cross-sectional survey in Makkah identified concerns regarding medication shortages and communication between patients and pharmacists (16), while in Al-Ahsa, 84.1% of patients reported being at least somewhat satisfied with Wasfaty services (9). Additionally, a study among community pharmacists in the Qassim region reported that almost three-fourths of respondents did not agree that the Wasfaty system had improved patient satisfaction, reflecting mixed perceptions regarding its effectiveness (17).

Potential determinants of patient satisfaction include sociodemographic characteristics (age, gender, education, occupation, nationality, marital status), purpose of pharmacy visit (acute vs. chronic conditions), pharmacy-related factors (availability of medications, waiting time, staff communication and professionalism, pharmacy environment), and Wasfaty-specific features (ease of use, frequency of use, and ability to obtain prescribed medications). Identifying these factors is crucial for enhancing healthcare services, optimizing the efficiency of the Wasfaty system, and ensuring patient adherence and safety, particularly as healthcare systems worldwide increasingly adopt digital health applications (18). However, despite the national implementation of Wasfaty, patient satisfaction with the system and associated community pharmacy services has not yet been evaluated in the Aseer region. While studies from Makkah and Al-Ahsa provide insights into factors affecting satisfaction, regional differences in healthcare infrastructure, pharmacy accessibility, and patient demographics may result in unique barriers or facilitators in Aseer. Evaluating patient satisfaction and its determinants locally is therefore essential to identify region-specific challenges, guide targeted interventions, and optimize the delivery of pharmacy services in the region (18). Accordingly, this study aimed to assess patient satisfaction with Wasfaty and community pharmacy services in the Aseer region of Saudi Arabia and to identify the sociodemographic, pharmacy-related, and Wasfaty-specific determinants influencing satisfaction.

## 2 Materials and methods

### 2.1 Study design

This cross-sectional study was conducted in the Aseer province of Saudi Arabia using an anonymous, self-administered online questionnaire, with data collected between 5 September and 25 October 2025.

### 2.2 Study population and eligibility criteria

Adult patients (≥18 years) who had previously used Wasfaty services were included in the study. Patients who had never used Wasfaty, were younger than 18 years, utilized private healthcare services in Saudi Arabia, or did not provide informed consent and incomplete responses were excluded.

### 2.3 Sample size and sampling procedure

**FIGURE 1:**
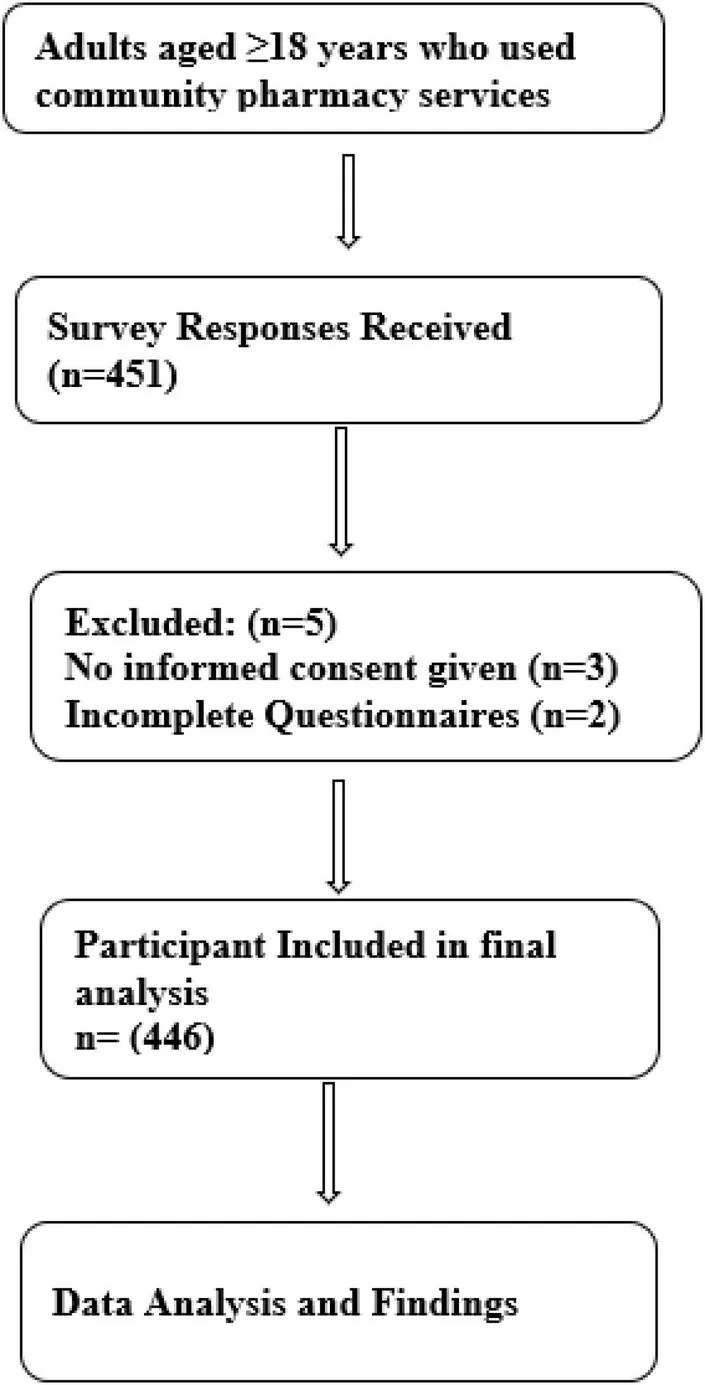
Flow diagram of participant recruitment and selection.

The sample size was calculated based on the estimated population of the Aseer region (2,024,285), obtained from the Saudi General Authority for Statistics (GASTAT) (19), using a 95% confidence level and a 5% margin of error. To enhance the study’s statistical power and to account for potential exclusions, data collection continued beyond the minimum target. A non-probability convenience sampling technique was employed. Data were collected from adults (≥18 years) at Ministry of Health Primary Health Care Centers (PHCs), where Wasfaty e-prescriptions are generated, and at community pharmacies contracted with the Wasfaty program, where these prescriptions are dispensed. To facilitate broader participation at these sites, a QR-code survey link was also displayed at PHCs and participating pharmacies, allowing users to complete the questionnaire electronically. However, this approach may inherently exclude individuals with limited digital access or low digital literacy, as well as non-users of Wasfaty services, which may affect the representativeness of the study population. This approach ensured inclusion of patients across different points of the Wasfaty service pathway; however, it may also introduce selection bias, as individuals who are more digitally literate, more engaged with healthcare services, or more satisfied with Wasfaty may have been more likely to participate. Therefore, the sample may not be fully representative of all Wasfaty users in the Aseer region, and the findings should be interpreted with caution. Participation was voluntary and anonymous. A total of 451 responses were received. Of these, 5 responses were excluded because participants did not provide consent (*n* = 3) or submitted incomplete responses (*n* = 2). Therefore, 446 valid responses were included in the final analysis (Figure 1).

### 2.4 Data collection instrument

A structured questionnaire, adapted from previous studies with similar objectives (16, 20), was used. It consisted of four sections: Section A: Sociodemographic information (gender, age, education, occupation, nationality, marital status, and purpose of the pharmacy visit). Section B: Experience with Wasfaty, including frequency of use, ease of use, and ability to obtain prescribed medications (3 items). Section C: Satisfaction with community pharmacy services, measured using a five-point Likert scale (1 = Very dissatisfied to 5 = Very satisfied), covering medication availability, pharmacist explanations, communication and professionalism, waiting time, pharmacy environment, and overall interaction with pharmacy staff (6 items). Section D: Assessed overall satisfaction with Wasfaty and community pharmacy services (2 items), and willingness to recommend these services to others (1 item). Wasfaty-related items were measured using a five-point Likert scale ranging from 1 (Very dissatisfied) to 5 (Very satisfied). The overall satisfaction score was computed as the mean of two items: (1) overall satisfaction with the Wasfaty experience and (2) overall satisfaction with community pharmacy healthcare services. The resulting score ranged from 1 to 5, with higher scores indicating greater satisfaction. The questionnaire was initially developed in English, translated into Arabic, and back-translated into English by experts to ensure accuracy. Face and content validity were evaluated by a panel of clinical pharmacy specialists. The online survey was created using Google Forms.

### 2.5 Pilot testing, reliability, and validity of the instrument

A pilot study was conducted with 30 participants to evaluate the clarity, comprehension, and completion time of the questionnaire. Based on feedback from the pilot study, revisions were made to improve item clarity and overall flow. Face validity was assessed during the pilot testing to ensure clarity and appropriateness of the items, while content validity was established through expert review by clinical pharmacy specialists. The refined questionnaire was then assessed for reliability and validity. Internal consistency of the satisfaction scale was confirmed with Cronbach’s alpha of 0.86. Sampling adequacy for inter-item analysis was assessed using the Kaiser–Meyer–Olkin (KMO) value of 0.80, and Bartlett’s test of sphericity was significant (*p* < 0.001), indicating that the data were suitable for assessing relationships among items.

### 2.6 Statistical analysis

Data were analyzed using SPSS version 26.0. Sociodemographic and background characteristics were summarized descriptively. Categorical variables were analyzed using Chi-square tests, while continuous variables were presented as mean ± SD. One-way analysis of variance (one-way ANOVA) was used to assess differences in mean scores across independent groups, including comparisons of experience and satisfaction scores between participants attending community pharmacies for acute and chronic conditions, as well as differences across categories of Wasfaty usage frequency. Likert-scale responses were treated as approximately continuous variables. Statistical significance was set at *p* < 0.05. Associations among categorical variables were examined using Chi-square tests with Cramer’s V. Likert-scale satisfaction items were treated as continuous variables for statistical analyses. Pearson correlation analysis was used to explore relationships between key satisfaction domains. Linearity assumption was assessed using scatterplots and partial regression plots, while multicollinearity was evaluated using variance inflation factor (VIF), with all variables showing values below 2, indicating no significant multicollinearity. Multiple linear regression analysis was performed to identify predictors of overall satisfaction with Wasfaty and community pharmacy services. The adequacy of the regression model was supported by model fit indices (*R*^2^ = 0.37, adjusted *R*^2^ = 0.35), and statistical significance was set at *p* < 0.05. Binary logistic regression analysis was then conducted to identify determinants of high overall satisfaction. For this analysis, the overall satisfaction score was dichotomized based on the 5-point Likert scale interpretation, where scores >4 were classified as “high satisfaction” (very satisfied), and scores of 4 or less were classified as “low to moderate satisfaction.” This classification was adopted to enhance interpretability and is consistent with the “top-box” approach used in patient-reported experience systems such as the Centers for Medicare & Medicaid Services (CMS) Hospital Consumer Assessment of Healthcare Providers and Systems (HCAHPS), where the highest response category defines optimal satisfaction for benchmarking purposes (21). Similar approaches have been reported in prior patient experience and healthcare quality studies (22–24). The sample size (*n* = 446) met regression guidelines of 10–20 observations per predictor variable. With eight predictors, this provided ∼56 observations per variable, exceeding recommended thresholds and indicating adequate model stability. Event-per-variable (EPV) assessment further supported sample adequacy for logistic regression. The logistic regression model included eight independent variables. Model adequacy was confirmed through the Hosmer–Lemeshow goodness-of-fit test (*p* = 0.52) and Nagelkerke *R*^2^ (0.36). Adjusted odds ratios (AORs) with 95% confidence intervals (CIs) were reported. Statistical significance was set at *p* < 0.05.

### 2.7 Ethical considerations

Ethical approval for this study was obtained from the Research Ethics Committee at King Khalid University (KKU-68-2025-20; HAPO-06-B-001). The first page of the survey included an informed consent statement outlining the study objectives, the voluntary nature of participation, and assurances of confidentiality. To ensure privacy, no personal identifiers were collected, and all data were obtained anonymously.

## 3 Results

A total of 446 individuals participated in the study. Females constituted 57.6% of the sample, while males accounted for 42.4%. The largest age group was 30–39 years (36.8%), followed by 40–49 years (26.9%), above 50 years (23.3%), and 18–29 years (13.0%). More than half of participants held a bachelor’s degree (56.1%), while others had completed high school (17.5%), diploma (13.9%), or postgraduate education (12.5%). Slightly more than half of the respondents were unemployed (52.9%), and the majority were Saudi nationals (98.1%). Most participants were married (63.9%). Regarding pharmacy visits, 70.4% visited for acute conditions, while 29.6% visited for chronic conditions (Table 1). Participants reported varied levels of engagement with the Wasfaty system. About 41.5% used it occasionally, while 33.8% were frequent users; a smaller group were first-time users (8.1%) or used it rarely (16.6%). When evaluating usability, most respondents rated the system positively, with 35.4% describing it as easy and 40.4% as very easy, whereas only 6.7% experienced difficulty. A statistically significant difference in satisfaction scores was observed across levels of ease of use [*F*(1, 444) = 4.72, *p* = 0.031]. Regarding medicine accessibility through Wasfaty, nearly 42.8% reported always receiving their prescribed medication, and 33.2% did so most of the time. Only a small proportion rarely or never obtained their medications (5.6%). Satisfaction with medication availability in pharmacies was also high, with 43.0% satisfied and 34.8% very satisfied. Pharmacist-related services were generally well-rated. For medication counseling, 43.9% of participants were satisfied and 35.9% were very satisfied. Similarly, communication and professionalism received strong ratings, with a combined 78.9% expressing satisfaction. Waiting times were considered acceptable, as 45.3% reported being satisfied and 31.4% very satisfied. Participants also viewed the pharmacy environment positively, with 44.2% satisfied and 37.0% very satisfied regarding cleanliness, comfort, and privacy. Overall interactions with pharmacy staff were well-received, with 44.4% satisfied and 35.8% very satisfied. General satisfaction with the Wasfaty experience including ease of use, access, and availability was high, with 40.8% satisfied and 36.7% very satisfied. Satisfaction with community pharmacy healthcare services followed a similar pattern, as 43.3% were satisfied and 36.1% very satisfied. Finally, willingness to recommend Wasfaty and community pharmacy services to others was strong: 68.1% indicated “yes,” while 24.7% expressed “maybe.” A chi-square test showed a significant association between recommendation preference and satisfaction levels (χ^2^ = 18.9, *p* < 0.001) (Table 2).

**TABLE 1:** Sociodemographic characteristics of participants.

Variable	Category	Frequency (n) *n* = 446	Percentage (%)
Gender	Male	189	42.4
	Female	257	57.6
Age (years)	18–29	58	13.0
	30–39	164	36.8
	40–49	120	26.9
	> 50	104	23.3
Education level	High school	78	17.5
	Diploma	62	13.9
	Bachelor’s degree	250	56.1
	Postgraduate	56	12.5
Occupation	Employed	210	47.1
	Unemployed	236	52.9
Nationality	Saudi	437	98.1
	Non-Saudi	9	1.9
Marital status	Single	161	36.1
	Married	285	63.9
Purpose of your pharmacy visit	Acute condition	314	70.4
	Chronic condition	132	29.6

**TABLE 2:** Participants’ experience and satisfaction with the Wasfaty system and community pharmacy services.

Variable	Response options	Frequency (*n*)	Percentage (%)	Mean ± SD	ANOVA *F*(df1, df2)	*P*-value
1. How often do you use the Wasfaty system?	Never used (first time user)	36	8.1	2.14 ± 1.06	*F*(3, 442) = 1.67	0.173
	Rarely	74	16.6			
	Occasionally	185	41.5			
	Frequently	151	33.8			
2. How would you rate the ease of using Wasfaty?	Very difficult	9	2.0	4.12 ± 0.86	*F*(1, 444) = 4.72	0.031*
	Difficult	21	4.7			
	Neutral	78	17.5			
	Easy	158	35.4			
	Very easy	180	40.4			
3. Were you able to obtain your prescribed medication through Wasfaty?	Never	8	1.8	4.05 ± 0.93	*F*(1, 444) = 0.65	0.42
	Rarely	17	3.8			
	Sometimes	82	18.4			
	Most of the time	148	33.2			
	Always	191	42.8			
4. Availability of medications in the pharmacy	Very dissatisfied	10	2.2	4.05 ± 0.93	*F*(1, 444) = 0.84	0.36
	Dissatisfied	18	4.0			
	Neutral	71	15.9			
	Satisfied	192	43.0			
	Very satisfied	155	34.9			
5. Pharmacist’s explanation of how to use your medication	Very dissatisfied	8	1.8	4.10 ± 0.85	*F*(1, 444) = 1.56	0.21
	Dissatisfied	13	2.9			
	Neutral	69	15.5			
	Satisfied	196	44.0			
	Very satisfied	160	35.8			
6. Pharmacist’s communication and professionalism	Very dissatisfied	6	1.3	4.07 ± 0.88	*F*(1, 444) = 1.23	0.27
	Dissatisfied	16	3.6			
	Neutral	72	16.1			
	Satisfied	196	44.0			
	Very satisfied	156	35.0			
7. Waiting time to receive medications	Very dissatisfied	7	1.6	4.08 ± 0.91	*F*(1, 444) = 0.95	0.33
	Dissatisfied	14	3.1			
	Neutral	83	18.6			
	Satisfied	202	45.3			
	Very satisfied	140	31.4			
8. Pharmacy environment (cleanliness, comfort, privacy)	Very dissatisfied	5	1.1	4.18 ± 0.87	*F*(1, 444) = 0.68	0.41
	Dissatisfied	11	2.5			
	Neutral	68	15.2			
	Satisfied	197	44.2			
	Very satisfied	165	37.0			
9. Overall interaction with pharmacy staff (including pharmacists and assistants)	Very dissatisfied	8	1.8	4.12 ± 0.89	*F*(1, 444) = 1.28	0.26
	Dissatisfied	15	3.4			
	Neutral	67	15.0			
	Satisfied	198	44.4			
	Very satisfied	158	35.4			
10. Overall, how satisfied are you with your Wasfaty experience (ease of use, access, and availability)?	Very dissatisfied	8	1.8	4.06 ± 0.92	*F*(1, 444) = 1.19	0.28
	Dissatisfied	19	4.3			
	Neutral	73	16.4			
	Satisfied	182	40.8			
	Very satisfied	164	36.7			
11. Overall, how satisfied are you with community pharmacy healthcare services?	Very dissatisfied	6	1.3	4.10 ± 0.90	*F*(1, 444) = 1.37	0.24
	Dissatisfied	17	3.8			
	Neutral	69	15.5			
	Satisfied	193	43.3			
	Very satisfied	161	36.1			
12. Would you recommend using Wasfaty and community pharmacies to others?	Yes	304	68.1	−	χ^2^(2) = 18.9	<0.001*
	No	32	7.2			
	May be	110	24.7			

Item 1 compares Wasfaty usage frequency across age groups (18–29, 30–39, 40–49, and > 50 years). Items 2–11 were analyzed using one-way analysis of variance (one-way ANOVA) to compare experience and satisfaction scores between participants attending community pharmacies for acute and chronic conditions. Likert-scale responses were treated as approximately continuous variables. Statistical significance was set at *p < 0.05.

Table 3 presents the correlation matrix among key variables related to the Wasfaty system and community pharmacy satisfaction. Ease of use demonstrated statistically significant moderate positive correlations with medication availability (*r* = 0.42, *p* < 0.001), pharmacist communication (*r* = 0.46, *p* < 0.001), pharmacy environment (*r* = 0.33, *p* < 0.001), overall Wasfaty satisfaction (*r* = 0.48, *p* < 0.001), and overall community pharmacy satisfaction (*r* = 0.44, *p* < 0.001). Medication availability showed moderate positive correlations with communication (*r* = 0.41, *p* < 0.001), pharmacy environment (*r* = 0.39, *p* < 0.001), overall Wasfaty satisfaction (*r* = 0.42, *p* < 0.001), and overall pharmacy satisfaction (*r* = 0.40, *p* < 0.001). Pharmacist communication was moderately correlated with overall Wasfaty satisfaction (*r* = 0.46, *p* < 0.001) and overall pharmacy satisfaction (*r* = 0.45, *p* < 0.001). The pharmacy environment also demonstrated moderate positive correlations with overall Wasfaty satisfaction (*r* = 0.39, *p* < 0.001) and overall pharmacy satisfaction (*r* = 0.37, *p* < 0.001). In contrast, waiting time showed weak negative correlations with ease of use (*r* = −0.18, *p* < 0.05), medication availability (*r* = −0.19, *p* < 0.01), communication (*r* = −0.15, *p* < 0.05), pharmacy environment (*r* = −0.22, *p* < 0.01), overall Wasfaty satisfaction (*r* = −0.21, *p* < 0.01), and overall pharmacy satisfaction (*r* = −0.19, *p* < 0.01). A strong positive correlation was observed between overall Wasfaty satisfaction and overall pharmacy satisfaction (*r* = 0.68, *p* < 0.001), indicating a strong association between these two variables.

**TABLE 3:** Correlation matrix of key variables.

Variable	Ease of use	Medication availability	Communication	Waiting time	Environment	Overall Wasfaty satisfaction	Overall pharmacy Satisfaction
Ease of use	1	0.42***	0.46***	−0.18*	0.33***	0.48***	0.44***
Medication availability		1	0.41***	−0.19**	0.39***	0.42***	0.40***
Communication			1	−0.15*	0.36***	0.46***	0.45***
Waiting time				1	−0.22**	−0.21**	−0.19**
Environment					1	0.39***	0.37***
Overall Wasfaty satisfaction						1	0.68***
Overall pharmacy satisfaction							1

*p < 0.05; **p < 0.01; ***p < 0.001.

Table 4 summarizes the associations between participant characteristics and overall satisfaction with the Wasfaty system. A statistically significant association was observed between gender and overall satisfaction (χ^2^ = 18.42, *p* < 0.001, Cramer’s *V* = 0.20), with a higher proportion of females reporting high satisfaction compared to males (83.2% vs. 61.4%). Education level was also significantly associated with satisfaction (χ^2^ = 9.12, *p* = 0.028, *V* = 0.14), with higher proportions of satisfaction observed among participants with bachelor’s and postgraduate education. No statistically significant differences were observed for age group, occupation, nationality, marital status, or purpose of visit (*p* > 0.05). Although participants aged 30–39 years showed the highest proportion of high satisfaction (77.3%), the association with age was not statistically significant (*p* = 0.15).

**TABLE 4:** Association between sociodemographic characteristics and overall Wasfaty satisfaction.

Variable	Category	High satisfaction (%)	χ^2^	df	*P*-value	Cramer’s V
Gender	Male	61.4	18.42	1	< 0.001	0.20
	Female	83.2				
Age (Years)	18–29	74.6	6.71	3	0.15	0.12
	30–39	77.3				
	40–49	72.0				
	> 50	68.4				
Education	High school	69.2	9.12	3	0.028	0.14
	Diploma	72.6				
	Bachelor’s	79.5				
	Postgraduate	82.1				
Occupation	Employed	78.4	3.94	1	0.14	0.09
	Unemployed	73.2				
Nationality	Saudi	77.4	0.21	1	0.65	0.02
	Non-Saudi	75.6				
Marital status	Single	74.5	2.58	1	0.28	0.08
	Married	78.1				
Purpose of visit	Acute condition	77.5	1.74	1	0.18	0.06
	Chronic condition	73.0				

Table 5 presents the multiple linear regression analysis examining predictors of overall satisfaction with Wasfaty and community pharmacy services. The model was statistically significant [*F*(7, 374) = 31.6, *p* < 0.001] and explained 37% of the variance in satisfaction (Adjusted *R*^2^ = 0.35). Gender was significantly associated with satisfaction, with females reporting higher levels compared to males (β = + 0.35, *p* < 0.001). Age showed a statistically significant but very small negative association with satisfaction (β = −0.02, *p* = 0.003). Among service-related variables, ease of using the Wasfaty system showed the strongest positive association with satisfaction (β = + 0.30, *p* < 0.001), followed by pharmacist communication (β = + 0.28, *p* < 0.001) and medication availability (β = + 0.24, *p* < 0.001). Waiting time showed a modest negative association with satisfaction (β = −0.10, *p* = 0.02). Education level (*p* = 0.15) and pharmacy environment (*p* = 0.33) were not significantly associated with satisfaction in the adjusted model. Variance inflation factor values ranged from 1.2 to 1.9, indicating no multicollinearity concerns.

**TABLE 5:** Multiple linear regression predicting overall satisfaction with Wasfaty and community pharmacy services.

Predictor variable	β Coefficient	Standard error	*T*-value	*P*-value	VIF
Gender (Female)	+ 0.35	0.08	4.12	< 0.001	1.4
Age	−0.02	0.01	−3.03	0.003	1.2
Education level	+ 0.06	0.04	1.45	0.15	1.3
Ease of using Wasfaty	+ 0.30	0.05	6.10	< 0.001	1.6
Medication availability	+ 0.24	0.05	4.90	< 0.001	1.8
Communication and professionalism	+ 0.28	0.05	5.20	< 0.001	1.9
Waiting time	−0.10	0.04	−2.34	0.02	1.5
Pharmacy environment	+ 0.04	0.04	0.97	0.33	1.7

Model summary: *R*^2^ = 0.37; Adjusted *R*^2^ = 0.35; *F*(7, 374) = 31.6; *p* < 0.001. VIF, Variance inflation factor.

**FIGURE 2:**
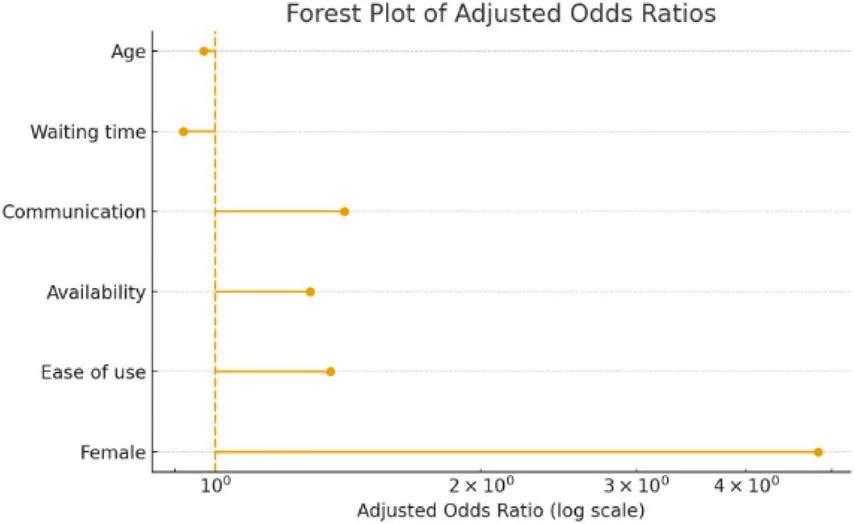
Adjusted odds ratios for factors associated with high patient satisfaction with Wasfaty services.

Table 6 presents the binary logistic regression analysis of factors associated with high overall satisfaction with the Wasfaty system and community pharmacy services. The model demonstrated acceptable fit, as indicated by the Hosmer–Lemeshow test (χ^2^ = 7.12, *p* = 0.52) and Nagelkerke *R*^2^ of 0.36, explaining 36% of the variance in satisfaction. Gender was significantly associated with higher odds of satisfaction, with females showing substantially greater odds compared to males (AOR = 4.83, 95% CI: 2.78–8.39, *p* < 0.001). Age showed a statistically significant but minimal negative association with satisfaction (AOR = 0.97, 95% CI: 0.95–0.99, *p* = 0.003). Among service-related factors, ease of using the Wasfaty system (AOR = 1.35, *p* < 0.001), medication availability (AOR = 1.28, *p* < 0.001), and pharmacist communication (AOR = 1.40, *p* < 0.001) were positively associated with higher odds of satisfaction. Waiting time was negatively associated with satisfaction (AOR = 0.92, *p* = 0.034). Education level (*p* = 0.34) and pharmacy environment (*p* = 0.20) were not significantly associated with satisfaction in the adjusted model (Figure 2). Overall, the findings indicate that user experience, staff communication, and medication accessibility are the most influential factors driving high satisfaction with the Wasfaty system, while demographic factors play a lesser but notable role.

**TABLE 6:** Binary logistic regression: to understand the odds ratio of various determinants of high overall satisfaction.

Predictor variable	Adjusted odds ratio (AOR)	95% Confidence interval	*P*-value
Gender (Female vs. Male)	4.83	2.78–8.39	< 0.001
Age (> 39 vs. ≤ 39 years)	0.97	0.95–0.99	0.003
Education (Bachelor’s + vs. ≤ Diploma)	1.12	0.89–1.41	0.34
Ease of using Wasfaty (per point)	1.35	1.18–1.55	< 0.001
Medication availability (per point)	1.28	1.11–1.47	< 0.001
Communication (per point)	1.40	1.20–1.63	< 0.001
Waiting time (per point)	0.92	0.85–0.99	0.034
Pharmacy environment (per point)	1.09	0.96–1.24	0.20

Model fit: Hosmer–Lemeshow χ^2^ = 7.12, *p* = 0.52; Nagelkerke *R*^2^ = 0.36.

## 4 Discussion

This study evaluated patient satisfaction with the Wasfaty e-prescription system and community pharmacy services in the Aseer region and found overall satisfaction to be high. However, responses were not uniformly positive across all domains, as a proportion of participants reported neutral or moderate levels of satisfaction in certain aspects of service experience. Most participants described the system as easy or very easy to use, reported reliable access to prescribed medicines, and expressed satisfaction with pharmacist communication, professionalism, and the pharmacy environment. Nearly three-quarters were willing to recommend Wasfaty to others. These findings closely mirror earlier Saudi studies in which approximately 70% of users reported positive experiences with Wasfaty and align with a recent national evaluation of 66,541 users showing satisfaction levels exceeding 70% across multiple domains (16). Similar patterns were observed in a regional study from the Jazan Region, demonstrating improved accessibility and more convenient service hours following the shift to community pharmacy–based dispensing (25).

Comparison with the Tabuk study reveals important demographic differences. While our sample consisted primarily of individuals aged 30–49 years, the Tabuk cohort was predominantly younger (18–30 years) (21). This difference may partly explain the higher proportion of acute-condition visits in our study (70.4%). Both studies, however, reported a predominance of Saudi nationals and individuals with bachelor’s degrees (20, 26), suggesting that Wasfaty users tend to be digitally literate. Prior studies have emphasized that individuals with lower education or limited digital experience may require tailored support to navigate e-prescription systems effectively (26). Additionally, national data show higher satisfaction among women and older adults (16), consistent with our findings, although age demonstrated a modest negative association with satisfaction in adjusted models.

Within the demographic analysis, female participants reported significantly higher satisfaction than male participants. This difference may reflect gender-based variations in healthcare expectations and service appraisal, particularly regarding interpersonal aspects of care such as communication and empathy. Previous literature indicates that female patients tend to place greater emphasis on relational components of healthcare when evaluating satisfaction (27). Furthermore, evidence from systematic reviews and experimental studies shows that practitioner empathy and effective communication are associated with improved patient satisfaction across healthcare settings (28, 29). In the context of the Wasfaty system and community pharmacy services, these interpersonal dimensions—particularly pharmacist communication, counseling, and patient engagement—may help explain the higher satisfaction observed among female users.

Our findings reinforce previous evidence showing strong satisfaction with operational aspects of Wasfaty, including ease of system use, medication availability, and pharmacist–patient communication (23, 30). Ease of use, communication quality, and medication accessibility were the strongest predictors of satisfaction across all analytic models. Comparison of effect sizes showed that ease of use (β = 0.30) had the largest positive association with satisfaction, followed closely by pharmacist communication (β = 0.28) and medication availability (β = 0.24). This pattern was consistent in the logistic regression analysis, where pharmacist communication (AOR = 1.40), ease of use (AOR = 1.35), and medication availability (AOR = 1.28) were associated with higher odds of satisfaction, whereas waiting time showed a comparatively smaller negative effect (AOR = 0.92). Moreover, correlations between these service domains and satisfaction were moderate (*r* = 0.33–0.48), while the relationship between overall Wasfaty satisfaction and overall community pharmacy satisfaction was substantially stronger (*r* = 0.68). Together, these findings indicate that service-related factors exert greater influence on satisfaction than demographic characteristics. These results are consistent with national and international research demonstrating the central role of pharmacist counseling, communication clarity, and medicine access in shaping patient satisfaction (30, 31). The national Wasfaty evaluation by Rasheed et al. (32) similarly highlighted improved therapeutic relationships and convenience related to prescription renewal and medicine refills. Waiting time has been identified as a key concern affecting patient satisfaction, with patients reporting service delays during the transition of pharmaceutical care services to community pharmacies in Saudi Arabia (33). Similar evidence has been reported in e-prescribing and digital pharmacy systems, where operational delays, longer prescription processing times, and workflow inefficiencies have been identified as key barriers affecting service efficiency and patient experience (34).

Increased demand at community pharmacies participating in the Wasfaty system, where medications are dispensed free of charge, may contribute to higher prescription volumes and workflow pressure, particularly during peak hours (24, 30, 35). Additionally, repeated concerns regarding unavailability of medicines, out-of-stock situations, and the need to visit multiple pharmacies have been widely reported in Saudi studies (36, 37). Earlier evaluations of community pharmacy practice have also emphasized persistent operational challenges, including medication shortage issues and variability in pharmacy preparedness (38, 39). These factors collectively highlight the need for stronger medication supply chains and better inventory management. Participants in the national survey also emphasized the need to expand medicine coverage, improve medication availability, and enhance advanced pharmacy services within the Wasfaty system (32).

Broader national evidence adds further context. Higher satisfaction has been reported in PHC pharmacies than in community pharmacies connected to Wasfaty, primarily due to communication challenges and limited interprofessional collaboration (40). Reviews consistently identify medication availability issues as one of the most persistent challenges in private pharmacy settings (41). Workforce characteristics may also play a role: over 90% of community pharmacists in Saudi Arabia are non-Saudis (42), which may contribute to communication barriers. Although expanding the Saudi pharmacist workforce has been recommended (42), many students prefer hospital-based practice (10), complicating nationalization efforts. Broader medication shortages driven by inadequate early-notification systems, low profit margins for essential medicines, and limited regulatory enforcement further contribute to inconsistent availability (35). Strengthening local pharmaceutical manufacturing capacity has been proposed as a long-term strategy to enhance medication security (43). The national assessment (32) further emphasized the importance of expanding medicine coverage and therapeutic options within the Wasfaty service to ensure broader access to treatment.

Demographic findings from our study provide additional insights. Females reported significantly higher satisfaction than males, consistent with previous studies where women rated communication quality and pharmacy environment more favorably (44, 45). However, the magnitude of the gender association was only moderate (Cramer’s *V* = 0.20), suggesting that although gender contributes to satisfaction differences, its influence is smaller than that of key service-related factors. Similarly, education demonstrated only a small effect size (*V* = 0.14) and was not retained as a significant predictor in multivariable analyses. These findings differ from studies in Pakistan where women reported lower satisfaction, possibly due to reduced comfort in seeking medication information (46). This contrast underscores how cultural norms influence communication dynamics across regions. Although education showed significant associations in univariate analysis, the effect was not retained in multivariable modeling, suggesting that service-related characteristics are more influential than demographic attributes in determining satisfaction. This differs from national studies where age, sex, and nationality were associated with satisfaction (44, 45), indicating potential regional differences in service delivery and workflow efficiency.

These findings suggest that while demographic factors such as gender may influence satisfaction levels, the overall variation in responses indicates that service experience is not uniform across all user groups, with some participants reporting neutral or less favorable perceptions of certain service domains. Beyond specific findings, this study holds broader implications for healthcare systems. The ongoing digital transformation in prescription services including electronic prescribing, online renewal, and digital refilling has the potential to reduce patient waiting times, enhance service efficiency, and improve overall satisfaction. Consistent with our findings and several related studies (47–53), community pharmacies play an increasingly vital role in delivering accessible pharmaceutical services. Successful implementation of systems like Wasfaty requires coordinated planning, adequate pharmacy participation, strong medication supply chains, and sufficient pharmacist staffing to deliver patient-centered care. Ensuring these elements may help facilitate smoother transitions from PHC-based dispensing to a sustainable community pharmacy–driven model.

Taken together, this study emphasizes the importance of prioritizing modifiable service components to enhance satisfaction. Reducing waiting times, improving medication availability, strengthening pharmacist counseling, and optimizing system integration should be focal points for improvement. Previous recommendations such as enhancing consultation privacy, maintaining medication records, and expanding preventive and monitoring services in pharmacies remain relevant (54). International experience from Finland, Spain, and European countries shows that mature e-prescription systems can achieve high satisfaction through clear information flow, minimal dispensing issues, and reduced medication errors (55, 56). Findings from patient satisfaction evaluations of the Wasfaty e-prescription system highlight areas for improvement, particularly in service quality, pharmacy workflow efficiency, and communication between pharmacists and patients (57). Overall, this study contributes to growing evidence that patient satisfaction with Wasfaty and community pharmacy services is primarily driven by usability, communication quality, and reliable medication access. However, variability in responses suggests that improvements are still needed to ensure more consistent service experiences across all patient groups. Comparison of effect sizes across correlation, regression, and association analyses consistently showed that service-related factors exerted larger effects on satisfaction than demographic characteristics. Ease of use, pharmacist communication, and medication availability demonstrated consistently moderate and practically meaningful associations with satisfaction, whereas demographic variables generally showed small-to-moderate effects. Demographic factors exert less influence once service-related variables are considered. Regional differences within Saudi Arabia highlight the need for context-specific strategies to ensure consistent, equitable, high-quality e-prescription and pharmacy services nationwide.

### 4.1 Limitations

This study has several limitations that should be acknowledged. First, the cross-sectional design captures patient satisfaction at a single point in time and does not allow for assessing changes as the Wasfaty system continues to evolve. Future longitudinal studies would help track satisfaction trends and better understand factors influencing patient experiences over time. In addition, due to its cross-sectional nature, causal relationships between variables cannot be established, and only associations can be inferred. Second, the study included only individuals who used the Wasfaty e-prescription system and collected their medications from contracted community pharmacies. Patients who preferred receiving their medications directly from PHC pharmacies, who were unable to use Wasfaty, or who faced barriers completing the process were not captured. This may introduce selection bias, as the experiences of non-Wasfaty users could differ substantially. In addition, a potential Hawthorne effect cannot be excluded, as participants were aware that they were being evaluated for a healthcare service, which may have influenced them to report more favorable responses than their actual experience. Third, data were collected using a QR-code–based digital questionnaire distributed at primary health centers and community pharmacies. This approach may have excluded individuals who are less comfortable with smartphone technology, have limited digital literacy, or have reduced access to digital health platforms. As a result, patients with lower digital engagement may be systematically underrepresented. In addition, QR-code–based recruitment may introduce participation bias, whereby individuals who are more digitally literate or more satisfied with the service are more likely to respond. Together, these factors may lead to an overestimation of satisfaction levels and limit the generalizability of the findings to the broader Wasfaty user population.

Fourth, the study relied on self-reported responses, which may be affected by recall bias or social desirability bias. Satisfaction levels might have been over- or underreported depending on personal expectations or past experiences. In particular, participants may have provided more favorable responses because they perceived the survey as being associated with healthcare providers or health services, thereby introducing social desirability bias and potentially inflating reported satisfaction levels. Fifth, only a limited number of demographic and service-related variables were assessed. Important factors such as socioeconomic status, health literacy, digital literacy, medication complexity, and severity of health conditions were not evaluated but may influence satisfaction with Wasfaty and community pharmacy services. Finally, some predictors included in the regression analysis—such as communication with pharmacists, medication availability, and waiting time—are conceptually closely related to components of the overall satisfaction construct. This may introduce conceptual overlap between independent and dependent variables, leading to partial circularity and potential inflation of observed associations. Therefore, the regression findings should be interpreted with caution. In addition, the study was conducted exclusively in the Aseer region. Variations in pharmacy workflows, medication availability, staffing patterns, and Wasfaty implementation across other regions of Saudi Arabia may limit the generalizability of the findings.

### 4.2 Future directions and implications

Future research should build on these findings by examining patient satisfaction over time, exploring patient perspectives through qualitative methods, and comparing experiences across different regions of Saudi Arabia. Further work assessing variations in pharmacy performance and incorporating digital health evaluation tools may provide deeper insights into factors influencing the usability and effectiveness of the Wasfaty system. Studies evaluating the impact of pharmacist training, workflow optimization, and supply-chain improvements would also help identify practical strategies to enhance satisfaction.

From a policy and practice standpoint, ensuring consistent access to prescribed medications is a priority, as gaps in availability contributed to neutral or dissatisfied responses. Efforts to reduce waiting times, standardize communication quality across pharmacies, and improve the user-friendliness of the Wasfaty platform particularly for first-time or older users may further strengthen patient experiences. Targeted quality improvement initiatives in lower-performing settings and data-driven decision-making could support more consistent service delivery and enhance the overall performance of the Wasfaty and community pharmacy services.

## 5 Conclusion

This study demonstrates that patients in the Aseer region generally report high satisfaction with the Wasfaty system and community pharmacy services, reflecting strengths in ease of system use, medication access for most users, and pharmacist communication. These positive experiences indicate that the integrated digital prescription service is functioning effectively for a large proportion of patients. At the same time, the results show that satisfaction is not uniform across all domains. A noticeable minority of participants expressed neutral or dissatisfied responses particularly regarding medication availability, waiting times, and certain aspects of staff communication. While these proportions were relatively smaller, they highlight areas where patient experiences can still be enhanced. Ensuring consistent access to prescribed medicines, minimizing delays in service, and maintaining high communication standards across all pharmacies may help improve overall satisfaction. Taken together, the findings present a largely positive picture of Wasfaty and community pharmacy performance, while also identifying practical opportunities for quality improvement. Strengthening these aspects may further enhance patient trust, service consistency, and the overall effectiveness of digital prescription and pharmacy services in the region.

## Data Availability

The original contributions presented in this study are included in this article/supplementary material, further inquiries can be directed to the corresponding author.
