# Correction: Determinants of patient satisfaction with Wasfaty and community pharmacy services in Aseer, Saudi Arabia: a cross-sectional study

**DOI:** 10.3389/fmed.2026.1947087

**Published:** 2026-08-25

**Authors:** Geetha Kandasamy, Khalid Orayj, Eman Shorog, Rayah Asiri, Tahani Alanazi, Asma M. Alshahrani, Amjad Hmlan, Ayesha Siddiqua, Hanan S. Algorman

**Affiliations:** 1 Department of Clinical Pharmacy, College of Pharmacy, King Khalid University, Abha, Saudi Arabia; 2 Department of Clinical Pharmacy, College of Pharmacy, Shaqra University, Dawadimi, Saudi Arabia

**Keywords:** community pharmacy services, digital prescription system, medication availability, patient satisfaction, Saudi Arabia, Wasfaty

An incorrect grant number was provided for King Khalid University's Research Project. The correct number is RGP 2/45/47.

The original version of this article has been updated.

